# Quantum Dot-Based Optical Fiber Sensor for Flow Velocity Sensing at Low Initial Temperatures

**DOI:** 10.3390/s25072079

**Published:** 2025-03-26

**Authors:** Lei Sun, Yekun Cao, Rui Zhou, Min Li, Xiaoyan Wen, Ming-Yu Li, Shuo Deng, Sisi Liu, Haifei Lu

**Affiliations:** Department of Physics, School of Physics and Mechanics, Wuhan University of Technology, Wuhan 430070, China; sl2000@whut.edu.cn (L.S.); 298855@whut.edu.cn (Y.C.); 261254@whut.edu.cn (R.Z.); minli@whut.edu.cn (M.L.); wenxy@whut.edu.cn (X.W.); mingyuli.oliver@gmail.com (M.-Y.L.); dengshuo1990@whut.edu.cn (S.D.); liusisi0109@hotmail.com (S.L.)

**Keywords:** Fabry-Perot interferometer, photothermal effect, flow velocity, PbS QDs, micro-flowmeter

## Abstract

The accurate monitoring of flow velocity is crucial in applications such as blood microcirculation and microfluidic systems. However, the high sensitivity of current hot wire flowmeters is often achieved at the expense of increasing the initial temperature, which imposes significant limitations when measuring blood or other temperature sensitive fluids. In this study, a fiber sensor probe with a plano-concave cavity, fabricated from a PbS quantum dots (QDs)-doped photoresist, is proposed for the sensitive flow velocity detection of microfluidics. In the proposed hot wire-based micro-flowmeter, the excitation laser (980 nm) is efficiently absorbed and converted into thermal energy, while minimally affecting the high-quality interference of the cavity at the C-band. The experimental results show that only a 3 °C increase in temperature is required for flow velocity monitoring, with a sensitivity of 7.7 pm/(mm/s) achieved within a linear response range of 3.82 mm/s to 16.72 mm/s. Additionally, an intensity interrogation scheme is introduced for the hot wire-based fiber sensor probe. This low initial temperature requirement makes the proposed sensor suitable for microfluidics, demonstrating promising potential for use in microcirculation measurement and drug delivery systems.

## 1. Introduction

In recent years, all optical flowmeters fabricated using optical fiber have garnered significant attention due to their notable advantages, such as a compact size, resistance to electromagnetic interference, high sensitivity, and ease of integration [1]. In contrast to fiber-optic flow velocity sensors that primarily rely on the stress exerted by flow on the optical fiber typically used for high flow velocity detection, the flowmeters based on dynamic thermal equilibrium, which involve heat exchange between the fluid and the sensor, have been widely adopted to meet diverse practical requirements [2]. There are two primary approaches for fabricating hot wire optical fiber flowmeters. One approach uses fiber Bragg grating (FBG) as the sensing element. In this configuration, pump light is coupled to the fiber cladding through a coupling structure, and optical absorption material deposited on the cladding undergoes photothermal conversion. The temperature of the FBG will increase to a specific value after thermal convection with the surrounding environment, which then changes with variations of the external flow velocity [3,4,5,6,7]. Flow velocity measurement is achieved by demodulating the spectral shift of FBG [8,9,10,11]. For instance, Tang et al. reported a cladding-etched FBG optical fiber hot wire flowmeter in 2023 [3], which achieved a measurement range of 0–17 m/s with a temperature increase of 260 °C. However, the separation between the photothermal conversion and sensing region in this structure leads to relatively long response times. Moreover, due to the relatively low temperature sensitivity of the Bragg grating, it requires a substantial temperature increase to achieve accurate flow velocity measurement. This limitation is particularly significant in liquid measurements, especially in the measurement of bio-related fluids. Another approach is the use of a Fabry-Perot (F-P) cavity as the sensing element [12,13,14,15,16]. Unlike the FBG-based design, the F-P cavity integrates both the heating and sensing modules, which improves the sensor’s response time and reduces the size of the sensing unit. For example, Liu et al. reported a silicon cavity Fabry-Perot (F-P) hot wire optical fiber flowmeter in 2016, which achieved an average sensitivity of 52.4 nm/(m/s) in the range of 1.5 mm/s to 12 mm/s [16]. However, similar to the FBG approach, the silicon cavity has relatively low temperature sensitivity, requiring a high initial temperature to achieve accurate flow velocity measurement. More recently, the F-P microcavity based on Fe_3_O_4_-PDMS, reported by Li et al. in 2024 [12], utilizes the high temperature sensitivity of PDMS. This design achieved flow velocity measurements in the range of 0.05 mm/s to 0.6 mm/s with a temperature increase of 8–9 °C, making it more suitable for bio-related flow velocity measurements [12].

Accurate flow velocity measurement is crucial in fields such as blood microcirculation monitoring and minimally invasive interventional surgeries [17]. Quantitative data on microvascular morphology and hemodynamics are of vital importance for studying normal tissue development, detecting disease processes such as cancer, atherosclerosis, and diabetes, and for developing treatment methods for spinal cord injury (SCI) [17,18,19,20,21,22]. For instance, peak blood flow velocity in arteries reaches 8.3 mm/s across the transverse direction of the spinal cord and 25.8 mm/s along the spinal cord. Monitoring blood flow velocity can help to quantify hemodynamics, contributing to the development of therapeutic strategies for SCI [20]. In cases of coronary artery stenosis caused by atherosclerosis, coronary blood flow velocity can decrease to 0–22 mm/s, leading to coronary heart disease. By monitoring the blood flow velocity directly, the functional severity of stenosis can be assessed [17,21,23]. For invasive sensors, it is crucial that the sensor temperature does not exceed safe limits to avoid affecting the blood, and the sensor must also have a sufficient measurement range. Existing hot wire optical fiber flowmeters are unable to fully meet the demands for measuring flow velocity in living organisms. Therefore, a hot wire optical fiber flowmeter with a low initial temperature—one that can measure flow velocity with only several degrees increase in temperature relative to blood temperature—is necessary.

In this study, a full optical hot wire micro-flowmeter sensor based on lead sulfide quantum dots (PbS QDs, synthesized in our laboratory with an average size of 2.8 nm and an absorption peak ranging from 820 nm to 1000 nm) is designed and fabricated at the tip of a single mode fiber. The sensor consists of two layers of gold thin films and a Fabry-Perot resonant cavity formed by a composite material of PbS QDs and ultraviolet (UV) curable adhesive. The sensor capitalizes on the photothermal properties of QDs and the thermally induced optical property variations of the UV-cured adhesive. With a temperature increase of only 3 °C, the sensor achieves a sensitivity of 7.7 pm/(mm/s) within the linear range of 3.82 mm/s to 16.72 mm/s. To further reduce the demodulation cost and enhance the integration of the flow velocity testing system, flow velocity test data are also obtained through intensity demodulation in this study. The sensor’s micron-sized probe and low initial temperature make it well-suited for providing quantitative information regarding microvascular morphology and hemodynamics.

## 2. Principle

**Figure 1:**
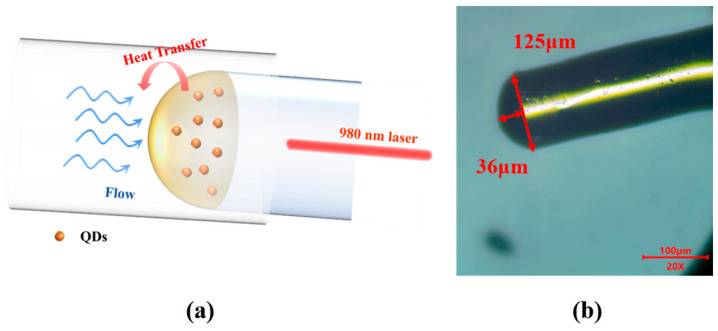
(**a**) Schematic illumination of the sensor probe. (**b**) Optical microscopic image of the sensor probe.

As shown in Figure 1a, the proposed flowmeter is a Fabry-Perot Interferometer (FPI) composed of two layers of gold films fabricated on the surfaces of single mode fiber (SMF, Yangtze Optical Fibre and Cable Joint Stock Limited Company, Wuhan, China) and the outer surface of cavity formed from a composite material of PbS QDs doped in UV-curable adhesive (Norland Optical Adhesive 61, Norland Products, Jamesburg, NJ, USA). Figure 1b, obtained by using an optical microscope (107JX, Shanghai Puda Optical Instrument CO, Shanghai, China) under 20× objective magnification, presents an optical microscopic image of the sensor, which has a cavity length of approximately 36 μm. Due to the size dependent bandgap of QDs, the pump light at 980 ± 8 nm is partially absorbed and converted into thermal energy, leading to an increase in the cavity temperature. For light with energy smaller than the bandgap of the QDs, the QD-doped adhesive acts as a non-absorptive medium, allowing strong interference between the two reflective gold mirrors. The interference wavelength can be expressed as follows [1]:

(1) $$\lambda_{m}=\frac{4\pi nL}{\left(2m+1\right)\pi },m=0,1,2,3\ldots$$

The interval between two adjacent troughs is defined as the Free Spectral Range (*FSR*) [7]:

(2) $$FSR=\frac{\lambda^{2}}{2nL}$$

where *λ*, *n*, and *L* represent the wavelength, the refractive index of the cavity material, and the cavity length, respectively.

During the sensing of liquid flow, the optical interference in the resonant cavity varies when the pump laser is incident, and it eventually stabilizes once thermal equilibrium is reached between the cavity and the external environment, such as at a fixed flow velocity. Changes in the external disrupt this equilibrium, leading to variations in the optical path length within the cavity, which in turn causes a spectral shift in the interference spectrum. Monitoring these spectral variations allows for the detection of changes in the external flow velocity. It is important to note that the spectral shift caused by the fluid pressure of flow velocity on the flowmeter can be ignored [12]. Therefore, the primary factor influencing the spectral shift is the heat exchange between the fluid and the sensor.

Heat transfer between the sensor and fluid follows the energy conservation law and Fourier’s heat conduction equation [1,24]:

(3) $$\left\{\begin{array}{l} \rho C_{p}\left(\frac{\partial T}{\partial t}+u\cdot \nabla T\right)+\nabla \cdot q=Q\\ q=-k\nabla T \end{array}\right.$$

In the above formula, *C_p_* represents the specific heat capacity (J/(kg⋅K)), *ρ* is the density of the object (kg/m^3^), *T* is the temperature (K), ***u*** is fluid velocity vector (m/s), *Q* is the heat flux per unit volume (W/m^3^), *q* is the heat exchange on the probe surface, and *k* is the thermal conductivity (W/(m⋅K)).

The proposed sensor is a hot wire sensor based on the principle of heat exchange. According to the classical heat transfer theory and Newton’s cooling formula, the fluid thermal convection equation is as follows [7,24,25,26]:

(4) $$H_{loss}=\left(A+Bv^{n}\right)\left(T_{v}-T_{0}\right)$$

where *T_v_* represents the temperature in the sensing probe, *T*_0_ represents the temperature of the external fluid, *v* is the fluid velocity, and *H_loss_* represents the heat loss induced by the fluid flow. When the fluctuations of the fluid temperature and physical property parameters are relatively small, *A* and *B* are defined as constants, which are mainly related to the structure of the probe.

When the thermal convection reaches equilibrium, it can be expressed as follows [7,26]:

(5) $$H_{loss}=P\varphi \alpha_{QDs-UV}$$

In the formula, *P* is the energy of pump laser injected into the fiber, *φ* is the coupling efficiency of the probe structure, and *α_QD__s__-UV_* is the optical absorption efficiency of the composite material. At thermal equilibrium, the temperature–flow velocity relationship is derived as follows [12]:

(6) $$T_{v}=\frac{P\varphi a_{QDs-UV}}{A+Bv^{n}}+T_{0}$$

The spectral shift caused by the cooling effect can be expressed as follows:

(7) $$\Delta \lambda_{T}=\frac{4n}{2m+1}\times L\times \beta_{neff}\times \frac{P\varphi a_{QDs-UV}}{A+Bv^{n}}$$

where $\beta_{neff}$ is the effective optical path variation induced by temperature change in the optical cavity, which is approximately 2.2 × 10^−4^ [1/K] [12].

## 3. Experiment and Discussion

A specific quantity of PbS quantum dots (QDs) in toluene is dispersed into a UV-curable adhesive. The mixture is stirred to achieve a homogeneous blend of the composite material, and a significant portion of the toluene is removed, resulting in a composite material with a quantum dot concentration of 10 mg/mL. Subsequently, the residual toluene and any bubbles in the solution are eliminated through vacuum degassing. A 12 nm gold film is deposited onto the polished end face of the optical fiber (with an inclination angle of less than 0.5°) via magnetron sputtering, serving as the first layer of the reflecting mirror. We suspend the optical fiber vertically and utilize a three-dimensional displacement platform to monitor the fiber’s end face during the application and curing of the composite material. An appropriate amount of the composite material is applied to the fiber end face using a 1 mL syringe. Under the influence of gravity and the liquid’s surface tension, a plano-concave cavity naturally forms on the fiber end face. After UV curing, a 100 nm gold film is magnetron sputtered onto the outer layer of the plano-concave cavity, acting as the second reflecting mirror. The manufacturing process of the sensor is relatively straightforward. However, controlling the cavity length remains challenging, which may affect the reproducibility of sensor fabrication. The gold film is deposited using physical vapor deposition (magnetron sputtering), ensuring a dense and durable layer that is resistant to peeling. The cavity, composed of UV-curable adhesive, exhibits strong adhesion, contributing to the sensor’s mechanical stability. Additionally, the stability can be further enhanced by sputtering a chromium adhesion layer between the gold film and the optical fiber.

**Figure 2:**
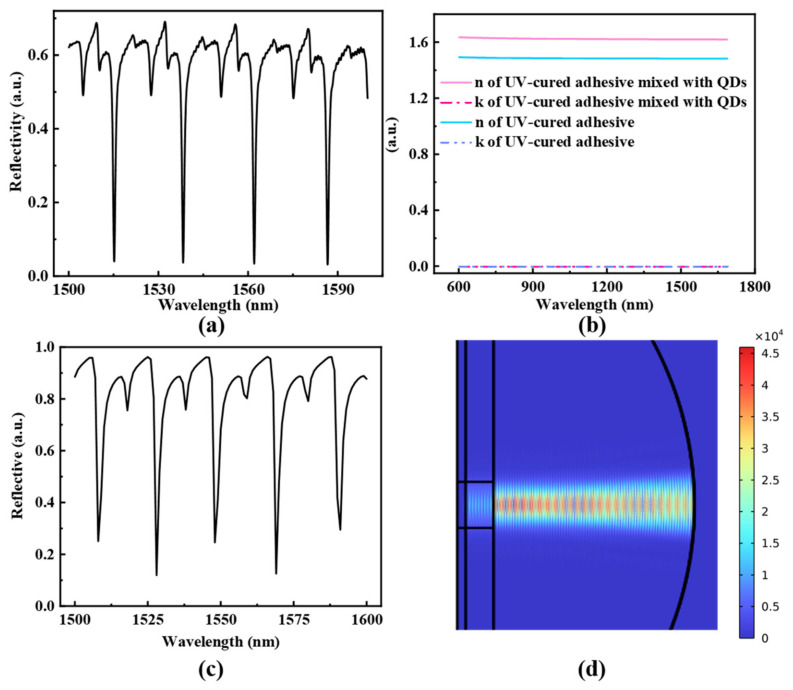
(**a**) Experimental reflection spectrum of the proposed fiber sensor probe. (**b**) The refractive index (n) and extinction coefficient (k) of UV-cured adhesive and PbS QDs-doped UV-cured adhesive. (**c**) Simulated reflection spectrum of plano-concave cavity prepared from PbS QDs-doped UV-cured adhesive. (**d**) The electric field distribution of the cavity at 1569 nm obtained by simulation.

The experimental inference spectrum of the prepared optical fiber sensor is shown in Figure 2a. The refractive index of the UV-cured adhesive, both before and after mixing with PbS QDs (10 mg/mL), is characterized using an ellipsometer, and the results are presented in Figure 2b. Based on the measured refractive index of the composite material and the captured profile of the concave mirror (Figure 1b), we utilized Finite Element Analysis software (COMSOL Multiphysics 5.3) and the theory of light transmission in a medium and optical interference to construct a simulation model. The simulated reflection spectrum of the sensor is shown in Figure 2c. The FSR of the plano-concave cavity can be calculated using Equation (2), yielding an approximate value of 22 nm, which is consistent with the simulation results. The electric field diagram at the resonant peak of 1569 nm, depicted in Figure 2d, confirms the strong interference within the optical cavity.

**Figure 3:**
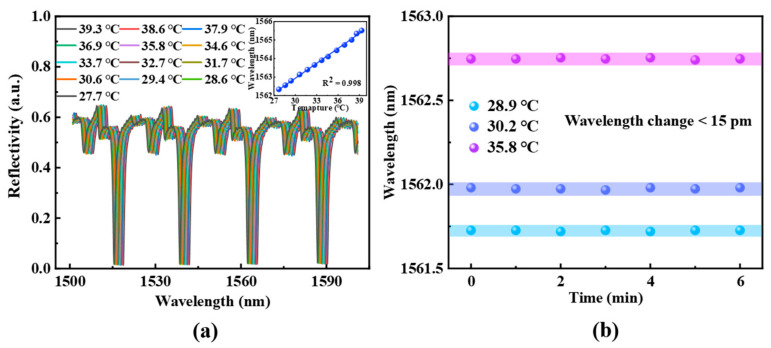
(**a**) Spectral response and linear fitting of temperature sensing characterization of the sensor probe. (**b**) Spectral stability test at the fixed temperature.

Before applying the proposed sensor to flow velocity detection, it is necessary to characterize its spectral response to temperature variations. The reflection spectrum is collected by placing the sensor in a temperature-controlled chamber, with the temperature range set to increase from 27.7 °C to 39.3 °C in approximately 1 °C increments. The spectral response of the optical fiber sensor to temperature changes is shown in Figure 3, demonstrating that the spectrum redshifts as the temperature increases. The temperature sensitivity, obtained through fitting, is 253 pm/°C, with a linearity of 0.998. Furthermore, the time-dependent optical response of the sensor at a fixed temperature is examined to verify its stability in temperature sensing. As illustrated in Figure 3b, three distinct temperatures are chosen for a 6 min stability test, with data recorded at 1 min intervals. These results confirm that the optical fiber probe exhibits favorable temperature stability.

**Figure 4:**
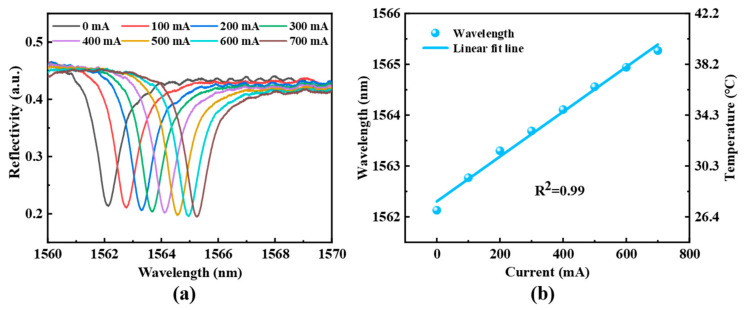
(**a**) Spectral response of the sensor probe under excitation of pump laser with different driven current, and (**b**) the fitting between spectral shift and driven current.

When a 980 nm pump light with energy at the absorption band edge of QDs is incident into the sensor probe, the photothermal effect of the PbS QDs causes the temperature of the resonant cavity to rise. Concurrently, due to the thermal expansion and thermo-optic effects of the UV-cured adhesive, the optical path within the sensing cavity increases, resulting in a spectral shift. To investigate the reflection spectrum of the optical fiber sensor under different pump light powers, tests are conducted while maintaining a constant ambient temperature and without flow velocity. The sensor’s response under varying optical powers is shown in Figure 4a. As the drive current for excitation laser increases, the interference spectrum undergoes a regular redshift, and a good linear relationship is observed in Figure 4b, indicating a direct correlation between the drive current and the temperature within the cavity.

**Figure 5:**
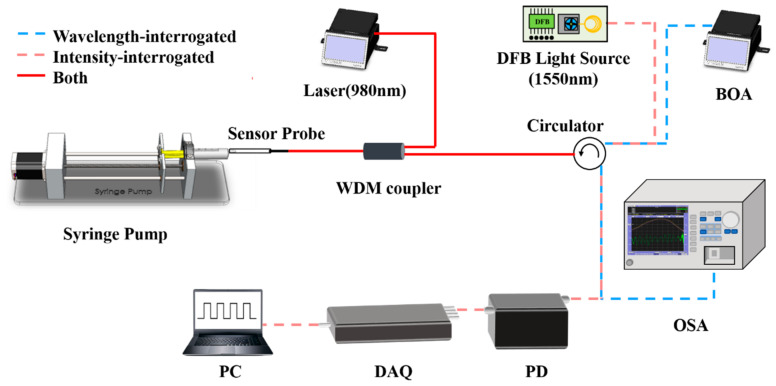
Schematic illustration of the fluidics flow rate measuring system.

To explore the sensor’s ability to detect flow velocity in liquid, optical systems based on both wavelength demodulation and intensity demodulation are constructed, as shown in Figure 5. The wavelength demodulation system consists of an optical fiber probe, a 980 nm pump laser (LUMENTUM, S27-7602-460, San Jose, CA, USA), a broadband light source (Booster Optical Amplifier, BOA, Thorlabs CLD1015, Newton, NJ, USA), an optical spectral analyzer (OSA, Yokogawa AQ6370C 600 nm–1700 nm, Houston, TX, USA), and a precision injection pump for the liquid. The broadband light source and the spectral analyzer are connected via a circulator, and a wavelength division multiplexer (WDM, 980 nm and 1550 nm) is used to transmit both the pump laser and the broadband light, facilitating effective wavelength demodulation. For the intensity demodulation setup, a 1550 nm Distributed Feedback Laser (DFB, Santur TL-2020-C-102A, Fremont, CA, USA) serves as the light source. Additionally, a photodetector (PD, Beijing Conquer Photonics KG-PR-500M, Beijing, China), a high-speed data acquisition card (DAQ, NI Cdaq-9171, Austin, TX, USA), and a personal computer (PC) are used for data acquisition and processing. During the flow velocity sensing experiment, the optical fiber probe is securely placed in a microfluidic channel. The microfluidic channel consists of a transparent rubber tube with a constant inner diameter of 2 mm, externally connected to a syringe for fluid flow. To prevent sensor displacement due to liquid buoyancy or flow, a support bracket and adhesive tape secure the sensor at the channel’s center. The liquid flow velocity is precisely controlled by a syringe pump with an electrical motor. We made a simple adiabatic environment using heat insulation materials to prevent the influence of external temperature changes on the experiment.

**Figure 6:**
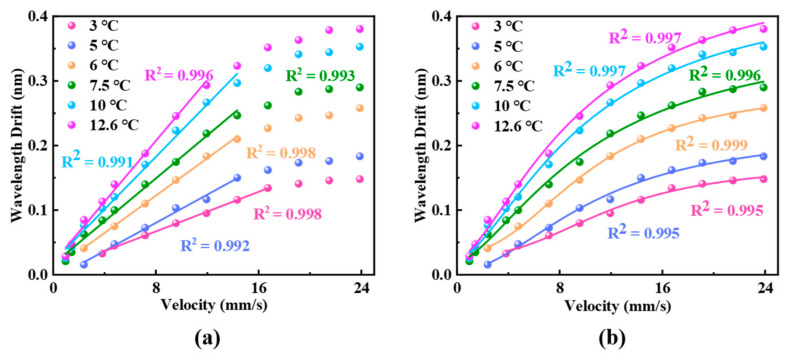
Experimental results of spectral drift corresponding with the variation in flow velocity. (**a**) Results of linear fitting and (**b**) the fitting results based on Equation (7).

Since we aim to reduce the initial temperature of the sensing probe for liquid flow detection, the drive current of pump laser is gradually increased to achieve a temperature rise of 3 °C, 5 °C, 6 °C, 7.5 °C, 10 °C, and 12.7 °C in the optical cavity. The interference valleys of reflection spectra corresponding to variations in flow velocity are shown in Figure 6. In the presence of liquid flow, heat generated in the optical cavity is partially dissipated, leading to a decrease in the temperature of the resonant cavity and an increase in spectral shift. At relatively low flow velocities, the sensor exhibits a linear relationship between the initial temperature and flow velocity. The sensitivities are fitted to be 7.7 pm/(mm/s), 10.9 pm/(mm/s), 14.45 pm/(mm/s), 16.63 pm/(mm/s), 20.3 pm/(mm/s), and 23.6 pm/(mm/s), respectively. The experimental results also show that higher initial temperatures enhance the sensitivity of the flowmeter, consistent with previous studies [3]. The linear sensing ranges of the flowmeter at different initial temperatures show minimal disparity. This is due to the relatively low initial temperatures during the testing procedure, which results in similar detection upper limits and linear ranges for the sensor. Compared with the fiber-optic thermal flow meters reported in the past two years, our sensor shows slightly lower sensitivity due to significant differences in the thermal expansion properties of the materials used. However, this also results in a unique flow velocity measurement range that does not overlap with other studies, effectively complementing intravascular flow velocity measurements.

**Figure 7:**
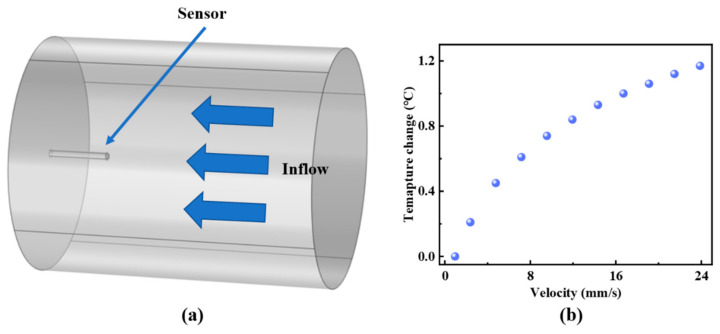
(**a**) Simulation model of flow velocity sensing. (**b**) Simulation results of sensor temperature variation with flow velocity.

Moreover, the spectral drift gradually increases and eventually levels off as the flow velocity increases. Figure 6b presents the fitting of the relationship between spectral shift and flow velocity according to Equation (7), demonstrating the consistency between theoretical and experimental results. For example, the fitting result is $y=0.171-\frac{0.899}{6.35+0.0098x^{2.61}}$ when the initial temperature is 3 °C; the others are in the same format as this fitting result. The gradual attenuation of sensitivity at higher flow velocities can be attributed to the nonlinear temperature variation within the optical cavity. Based on the fluid flow and heat transfer theory of Finite Element Analysis and Equation (3), Figure 7a shows the simulation model of temperature response of the sensor within the pipeline, with a temperature increase of 12.6 °C. The simulated temperature change in the sensor probe with varying flow velocity is illustrated in Figure 7b, which exhibits a trend similar to the experimental results in Figure 6.

**Figure 8:**
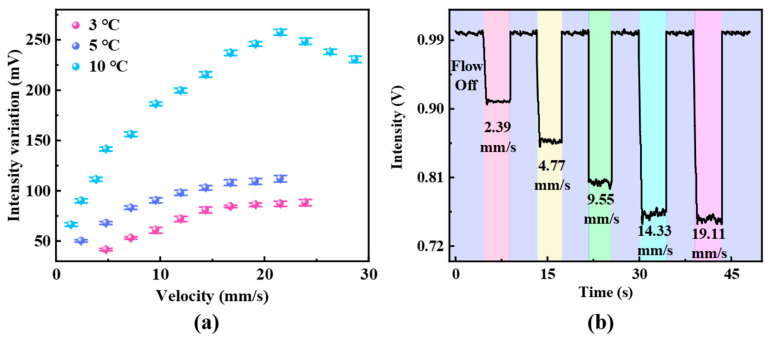
(**a**) Experimental results based on intensity demodulation for flow velocity sensing. (**b**) Dynamic response of the micro-flowmeter with different flow velocities.

Given that spectrometers are typically expensive and not suitable for integrated testing, this study also employed the intensity demodulation method. This method replaces the wideband light source and OSA with a DFB laser and photodetector, respectively, offering a cost-effective and easily integrated testing system. The variations in the average electrical intensity collected from the photodetector, under different initial temperatures, are documented within the flow velocity range of 0.96 mm/s to 23.88 mm/s. The experimental results based on the intensity demodulation scheme are presented in Figure 8a, where a general linear relationship is observed at relatively low flow velocities. By converting optical signal variations into electrical signals using a photodetector, demodulation is achieved. The data in Figure 8a represent the average values from multiple repeated experiments, corresponding to different flow velocities. By subtracting the electrical signal intensity at zero flow velocity, the final data in Figure 8a are obtained. For the demodulation result at a 10 °C temperature increase, the signal intensity initially increases then decreases as the flow velocity increases, which can be explained by the sensor’s working point. The working point is defined as the optimal wavelength of the demodulation light source that ensures the sensor’s measurement range and linear region. In this study, the working point is selected to be at a position on the descending edge of the reflection spectrum close to the interference peak. As the flow velocity increases, the working point moves out of the linear sensing range. Figure 8b shows the dynamic response of the electrical signal under different flow velocities with an initial temperature increase of 10 °C. In the absence of liquid flow, the electrical signal remains stable. However, when liquid flow is present, the electrical intensity shifts rapidly and stabilizes at a value corresponding to the flow velocity, confirming the feasibility of the intensity demodulation method. Although intensity demodulation is cost-effective and convenient, it still requires the determination of the operating point for each measurement. A proper strategy of swiftly determining the working point of the sensor would be necessary to improve the efficiency in the future.

## 4. Conclusions

In summary, we have developed a low-temperature, all-fiber hot wire micro-flowmeter that utilizes a PbS QDs-doped Fabry-Perot (F-P) cavity. The sensor enables precise flow velocity detection with a minimal temperature rise of only 3 °C, achieving a sensitivity of 7.7 pm/(mm/s) within a relevant flow velocity range (3.82 mm/s–16.72 mm/s). Additionally, the integration of intensity demodulation further enhances the system’s affordability and portability. This work addresses the critical need for biocompatible flow sensors, with potential applications in fields such as blood microcirculation monitoring and targeted drug delivery.

## Data Availability

The data are available from the corresponding author Haifei Lu upon reasonable request.
